# Different spatial distribution of inflammatory cells in the tumor microenvironment of ABC and GBC subgroups of diffuse large B cell lymphoma

**DOI:** 10.1007/s10238-021-00716-w

**Published:** 2021-05-06

**Authors:** Diego Guidolin, Roberto Tamma, Tiziana Annese, Cinzia Tortorella, Giuseppe Ingravallo, Francesco Gaudio, Tommasina Perrone, Pellegrino Musto, Giorgina Specchia, Domenico Ribatti

**Affiliations:** 1 Department of Neuroscience, Section of Anatomy, University of Padova, Padova, Italy; 2 Department of Basic Medical Sciences, Neurosciences, and Sensory Organs, University of Bari Medical School, Policlinico - Piazza G. Cesare, 11, 70124 Bari, Italy; 3 Department of Emergency and Transplantation, Pathology Section, University of Bari Medical School, Bari, Italy; 4 Department of Emergency and Transplantation, Hematology Section, University of Bari Medical School, Bari, Italy

**Keywords:** Diffuse large B cell lymphoma, Fractal dimension, Inflammatory cells, Spatial distribution, Tumor progression

## Abstract

Diffuse Large B-Cell Lymphoma (DLBCL) presents a high clinical and biological heterogeneity, and the tumor microenvironment chracteristics are important in its  progression. The aim of this study was to evaluate tumor T, B cells, macrophages and mast cells distribution in GBC and ABC DLBCL subgroups through a set of morphometric parameters allowing to provide a quantitative evaluation of the morphological features of the spatial patterns generated by these inflammatory cells.   Histological ABC and GCB samples were immunostained for CD4, CD8, CD68, CD 163, and tryptase in order to determine both percentage and position of positive cells in the tissue characterizing their spatial distribution. The results evidenced that cell patterns generated by CD4-, CD8-, CD68-, CD163- and tryptase-positive cell profiles exhibited a significantly higher uniformity index in ABC than in GCB subgroup. The positive-cell distributions appeared clustered in tissues from GCB, while in tissues from ABC such a feature was lower or absent. The combinations of spatial statistics-derived parameters can lead to better predictions of tumor cell infiltration than any classical morphometric method providing a more accurate description of the functional status of the tumor, useful for patient prognosis.

## Introduction

Diffuse Large B-Cell Lymphoma (DLBCL), classified by the 2008 WHO classification as one of the several types of large B-cell lymphomas, is the most common Non-Hodgkin Lymphoma (NHL), accounting for about 40% of all cases of NHL [1, 2]. DLBCL presents a high clinical and biological heterogeneity supported by the notion that most of these lymphomas arise from germinal center B-cells at different stages of differentiation, in which recurrent genetic alterations contribute to the molecular pathogenesis of the disease [3].

Gene expression profiling studies have contributed to unravel the complex biological and clinical heterogeneity of this disease, leading to the definition of a germinal center (GC) B-like DLBCL (GCB) subgroup and an activated B-like DLBCL (ABC subgroup), characterized by gene expression signatures such as constitutive activation of nuclear factor kB (NFkB) in ABC subgroup [4] and somatic mutations of polycomb repressor-2 complex gene EZH2 in GCB subgroup [5]. Moreover, the GCB subgroup expresses high levels of BCL6 and responds better to conventional chemotherapy, whereas the ABC subgroup has lower levels of BCL6 and tends to be refractory to chemotherapeutic treatment [6]. Moreover, we have demonstrated, comparing by means of RNA scope technology STAT-3 RNA expression in ABC and GBC subgroups, that ABC tissue sample contained a significant higher number of STAT-3 positive cells than GBC tissue samples [7]. STAT-3 is strongly linked to tumor angiogenesis and metastasis and is related to poor prognosis in different tumors [8].

Analysis of tumor microenvironment is an important aspect in the progression of DLBCL. Different cellular components of the tumor microenvironment, including macrophages and lymphocytes, have been considered in DLBCL such to establish correlations encompassing prognostic significance, stage-related tumor progression and differences in treatment outcome. We have previously demonstrated a positive correlation between STAT-3 expression and CD3-, CD8-, CD68- and CD-163 positive cells in the ABC and GBC subgroups [9].

The aim of this study is to determine the distribution of T and B cells, macrophages and mast cells distribution in GBC and ABC subtype DLBCL through a set of morphometric parameters allowing to provide a quantitative evaluation of the morphological features of the spatial patterns generated by these inflammatory cells, including size of the cell pattern,  shape of the cell pattern and architecture of the cell pattern.

## Materials and methods

### Patients

This retrospective study reviewed data from 30 patients diagnosed with DLBCL between 2015 and 2020. Tumors were divided into two histological subgroups: one that includes 15 ABC patients and another that includes 15 GCB patients.

### Immunohistochemistry

Paraffin-embedded tissues representatives of the DLBCL cases were sectioned at 3 μm. The sections were transferred onto poly-l-lysine-coated slides and subjected to deparaffinization and rehydratation. After blocking of endogenous peroxidases with a methanol-hydrogen peroxide solution for 30 min, a standard heat antigen retrieval in ethylene-diaminetetracetic acid (pH 8.0) was performed. The samples were then incubated with antibodies against CD4, CD8, CD68, CD 163, and tryptase (dilution 1:100, DAKO, Glostrup, Denmark). The sections were then incubated with biotinylated anti-mouse immunoglobulins, peroxidase-conjugated streptavidin and diaminobenzidine (DAB). Counterstain was performed with Harris hematoxylin. Each immunohistochemistry reaction was coupled with a positive control reaction (reactive lymph node) and a negative control reaction (no primary antibody).

### Slide scanning and analysis

For each case, three slides stained for CD4, CD8, CD68, CD163, and tryptase expression were scanned using the whole-slide scanning platform Aperio Scanscope CS (Leica Biosystems, Nussloch, Germany). All the slides were scanned at the maximum magnification available (40 ×) and stored as digital high-resolution images (TIFF file, 1712 × 1090 pixels) on the workstation associated with the instrument. Digital slides were inspected with the Aperio Imagescope v.11 software (Leica Biosystems, Nussloch, Germany) at 20 × magnification, and ten fields with equal area were selected for the analysis at 40 × magnification. CD4, CD8, CD68, CD163, and tryptase expression were assessed with the Positive Pixel Count algorithm embedded in the Aperio Imagescope software and reported as a percentage of positivity, defined as the number of positively stained pixels on the total of pixels of the image. Fields were selected in areas with the most intensive expression.

### Image analysis methods

**Fig. 1:**
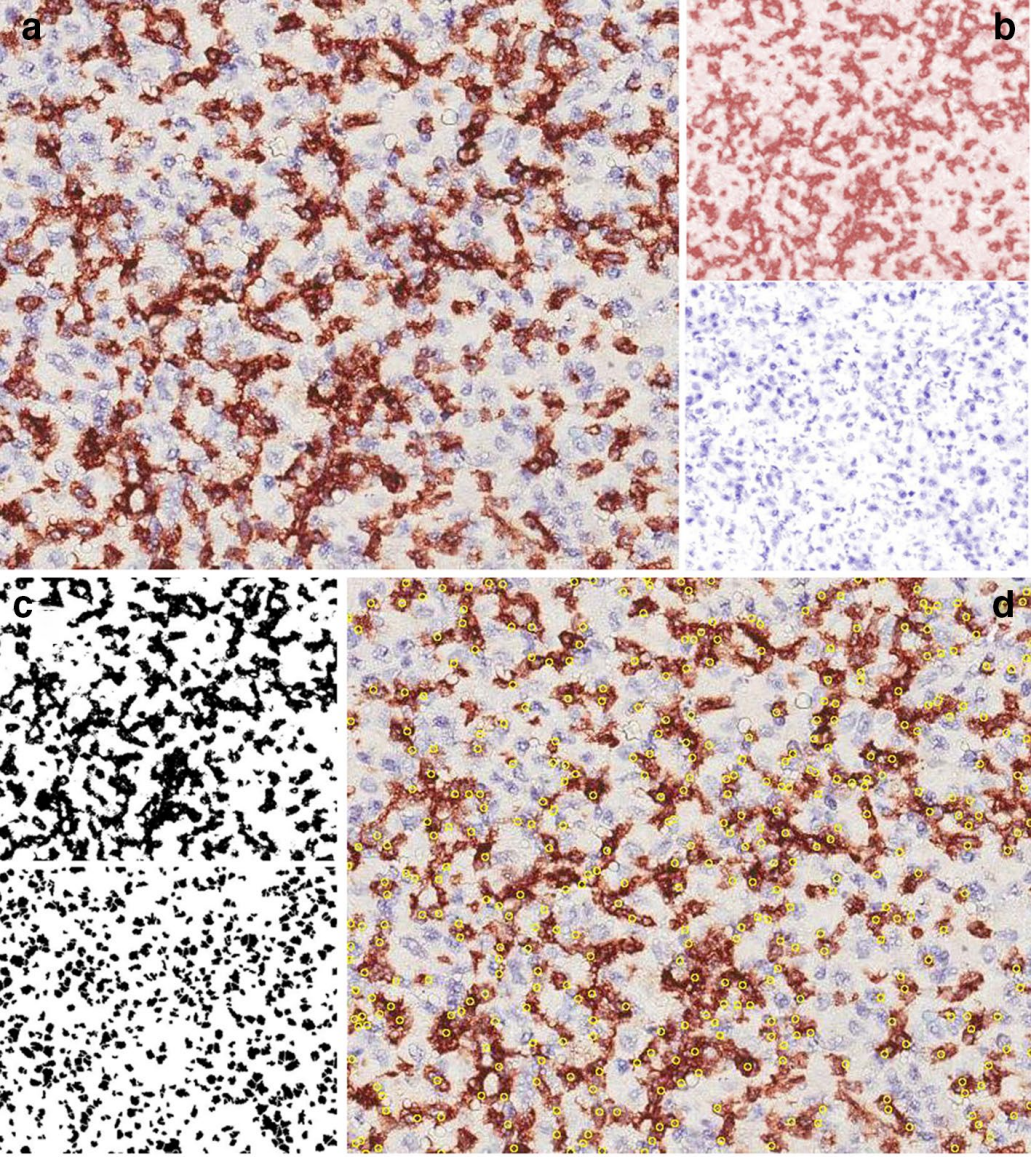
Schematic illustration of the image analysis procedure. **a** Microscope image from a tissue section processed for immunohistochemistry (anti-CD8 antibody, original magnification 40X). **b** Staining separation obtained by color deconvolution [11]: the DAB and the hematoxylin images are shown in the upper and lower panel, respectively. **c** From the images in **b** two binary images of the immunoreactive area (upper panel) and of the nuclei (lower panel) were obtained by conventional thresholding [12]. **d** By applying to the two binary images morphological and Boolean operations [28], the nuclei co-localized with [11] the immunoreactivity can be selected and their positions recorded. They appear as yellow circles superimposed to the original image

To estimate the immunoreactivity levels exhibited by the considered tissues, computer-assisted image analysis was performed by using the ImageJ software, freely available at http://rsb.info.nih.gov/ij/ [10]. Briefly, after shading correction and contrast enhancement, color deconvolution was applied. This procedure implements stain separation according to the method by Ruifrok et al. [11] and was performed by using an ImageJ plugin (named ‘Colourdeconvolution′) specifically developed by Gabriel Landini (see https://blog.bham.ac.uk/intellimic/g-landini-software/). This procedure leads to the generation of two images mainly containing DAB- and hematoxylin-stained structures, respectively. By conventional thresholding methods [12], immunoreactive structures can be easily discriminated from the first one, while the second one allows the discrimination of nuclear profiles (Fig. 1). The total amount of immunoreactive structures was evaluated by estimating the percent of tissue area (Area%) they occupy. The position of positive cells in the tissue was then obtained by selecting nuclear profiles co-localized with immunoreactive regions and estimating their (x,y) co-ordinates.

Starting from the coordinates, two methods were followed to characterize the spatial distribution of the positive cells in the tissues. The first one was a ‘morphological approach’ based on the estimation of a uniformity index according to a previously described procedure [13]. This index can have any value between 1 (when the objects are distributed in a regular array) and 0 (when maximal clustering occurs). The second one was an approach based on ‘spatial statistics’ (see [14] for details). It involves the calculation of the Ripley’s K-function [15] of the distances between cells. To interpret the cell-to-cell spatial relationship statistically, this function (K(d)) must be compared with the value estimated (K_0_(d)) on random (Poisson) point patterns. If K(d) is significantly greater than K_0_(d) for any range of d, then the cells are clustered, i.e., they are closer to each other than could be expected by chance. On the contrary, if K(d) is significantly lower than K_0_(d), then short cell–cell distances are less frequent than could be expected by chance, i.e., the placement of the cells exhibits ‘avoidance.’ For this reason, 100 random point patterns per analyzed field were computer generated. Each pattern had the same number of points as the number of observed cell profiles in the corresponding field.

### Statistics

Within each sample, the obtained immunoreactive area % and uniformity index values were averaged to provide a representative value of each parameter for that sample. Differences between GCB and ABC were then statistically tested by two-samples Student’s *t*-test. The GraphPad Prism 3.0 statistical package (GraphPad Software Inc., San Diego, CA) was used for the analysis, and *p* < 0.05 was considered as the limit for statistical significance. From the 100 random point patterns generated in association with each experimental pattern (see Image analysis methods), an estimate of the average K_0_-function together with its 95% confidence interval envelopes was obtained [14]. They were used to statistically assess deviations of the experimental K-function from randomness.

## Results

**Fig. 2:**
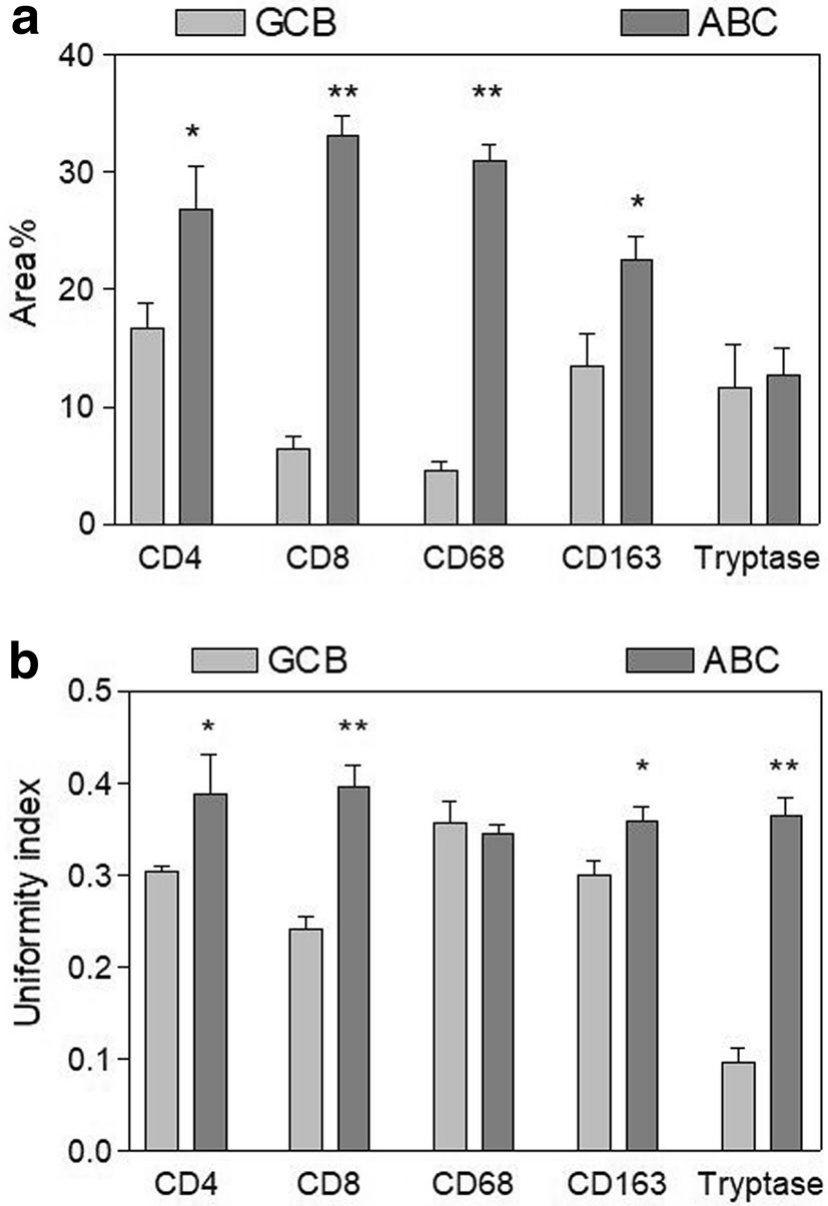
Morphometric analysis of the positive-cell patterns corresponding to the considered markers. Mean percent tissue area in **a** and uniformity index in **b** exhibited by the positive cells in tissue samples from GCB and ABC

As indicated by the immunoreactive area% values (Fig. 2a), the expression level of CD4, CD8, CD68 and CD163 was significantly higher in ABC tissue samples as compared to the samples from GCB, while no differences were detected between the two conditions in terms of the tryptase immunoreactivity.

The spatial distribution of positive cells, however, significantly differed between the two tissue types. As illustrated in Fig. 2b, the cell patterns generated by CD4-positive, CD8-positive, CD163-positive and tryptase-positive cell profiles exhibited a significantly higher uniformity index in ABC than in GCB tissue samples, indicating a tendency of the cells to assume a more uniform distribution in the tissues from ABC. The only exception was the distribution of CD68-positive cell-profiles which resulted similarly distributed in both ABC and GCB tissues.

**Fig. 3:**
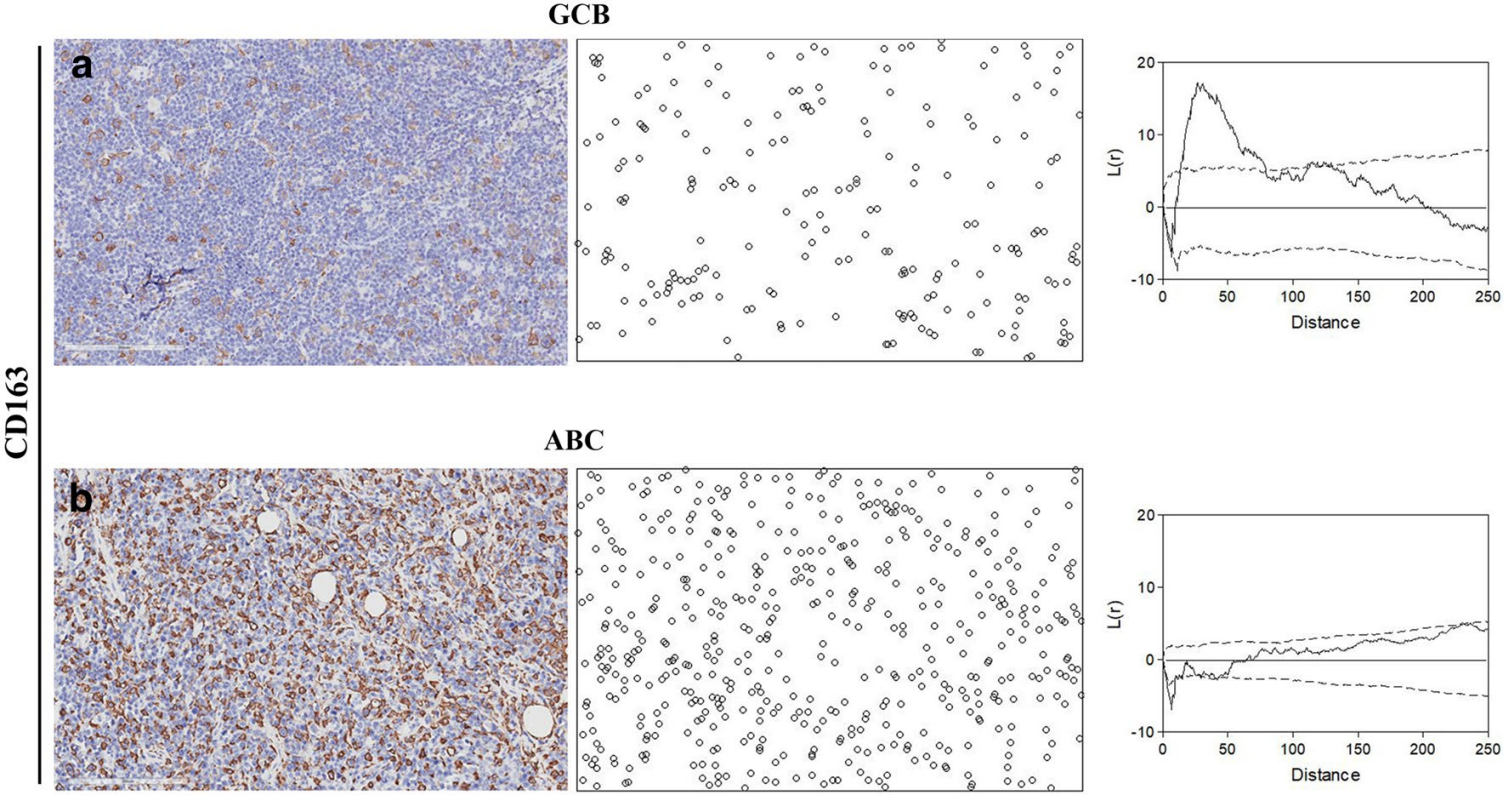
Spatial statistics-based analysis of the cell patterns from CD163-stained samples. Representative cell patterns from GCB in **a** and ABC in **b** tissue samples. Microscope images (left panel) are shown together with the corresponding pattern of positive cells (middle panel). In the right panel, the function L(d) = K(d)–K_0_(d) (see text) is plotted together with the upper- and lower-95% confidence interval envelopes for a random distribution (dashed lines). As shown, the L-function indicates that the CD163-positive cell pattern from GCB does not uniformly fill the tissue space but appears significantly aggregated since the L-function is outside the superior limit for a random point process for a quite large interval of cell-to-cell distances. On the contrary, the CD163-positive cell pattern from ABC does not differ significantly from a random spatial distribution

This finding was further confirmed by the analysis based on spatial statistics. For all the considered markers, indeed, the positive-cell distributions appeared clustered in tissues from GCB, as indicated by a K(d) significantly higher than the expected value for complete spatial randomness, while in tissues from ABC such a difference was lower or absent. An example is provided in Fig. 3 where data on CD163-positive cells are shown.

## Discussion

The inflammatory microenvironment has a strong impact on the development of cancer, which is seen not only as a genetic disease involving the accumulation of mutations that confer a selective advantage to the cancer cell but also as a result of the complex interactions and relationships that occur between immune cells, stromal cells, endothelial cells and the tumor cells.

Different inflammatory cells of the tumor microenvironment have been analyzed in DLBCL to establish different correlations regarding prognostic significance, stage-related tumor progression and differences in treatment outcome [16–19].

Our understanding of tumor heterogeneity in cancer progression derives from studies conducted at the genomic and transcriptomic levels, but little is known at the morphological distribution of the different cellular populations. We have already studied the pattern of distribution of mast cells in biopsy samples obtained endometrial adenocarcinoma, advanced primary melanoma, non-small lung carcinoma and cutaneous mastocytoma [13]. The results of this study have demonstrated that mast cells showed a virtual random spatial distribution, albeit with varying densities in all the tumors, despite histopathological differences.

The results of this study have evidenced that cell patterns generated by CD4-, CD8-, CD-68-, CD163- and tryptase-positive cell profiles exhibited a significantly higher uniformity index in the more aggressive ABC subtype than in the less aggressive GBC subtype, indicating a tendency of the cells to assume a more uniform distribution in the tissues from ABC. This finding was confirmed by the analysis based on spatial statistics. In fact, the positive-cell distributions appeared clustered in tissues from GCB, while in tissues from ABC such a feature was lower or absent.

The spatial distribution of the cells is of particular significance from the point of view of the biological processes occurring in tumor tissues and the development of tools able to quantitatively characterize the morphologic organization of the cell patterns could be of interest to study the biology of inflammatory cells in tumor microenvironment. The idea that tumors are composed of a heterogeneous tumor cell population suggests that the biological behavior of tumors depends on the composition of the tumor tissue rather than on a common characteristic of the tumor cell. In this context, it is extremely important to evaluate the characteristic of the different inflammatory cells constituting the tumor microenvironment. Different mathematical models of cancer have been developed, but only recently scientists have recognized their value. In this respect, the spatial organization of the different components of this microenvironment emerged as a factor of particular interest. This feature, indeed, may significantly influence the interactions between the different cell populations as suggested by models of tissue growth [20] and signal diffusion in tissues [21]. Available simulation results indicated that cell distribution is a key factor affecting the diffusion of signals, growth factors and nutrients [21], which in turn impact on tissue growth and on the characteristics of the grown tissue pattern [20]. In this context, an almost uniform distribution of the signaling cells has been shown to be more efficient in promoting trophic effects [21]. The here reported results appear consistent with this suggestion.

From an experimental standpoint, fractal dimension has been used to quantitatively characterize the irregular morphology of tumors [22] and vasculature [23], and digital pathology has provided several opportunities [24] to estimate quantitative parameters of the involved cell populations by improving and automating tasks such as cell counting [25] and cell position mapping [26].

However, there is strong evidence of ecological phenomena occurring in the tumor microenvironment, and morphological descriptors characterizing tissue architecture and the relationship between the distributions exhibited by the different cell populations involved may be of significant help in the study of these phenomena. In this respect, the introduction of spatial statistics tools can provide a substantial support to this type of investigation by providing metrics (such as the here used uniformity index) to further expand the description of the complex structure of this tissue environment. As recently documented in a methodological study [27] based on CD68-positive macrophages within human head and neck tumors, combinations of spatial statistics-derived parameters can lead to better predictions of macrophage infiltration than any classical morphometric method. This type of approach, therefore, may provide a more accurate description of the functional status of the tumor, potentially leading to a better view on patient prognosis [27].
